# Computational Design of Epitope-Enriched HIV-1 Gag Antigens with Preserved Structure and Function for Induction of Broad CD8^+^ T Cell Responses

**DOI:** 10.1038/s41598-018-29435-1

**Published:** 2018-07-26

**Authors:** Benedikt Asbach, Johannes P. Meier, Matthias Pfeifer, Josef Köstler, Ralf Wagner

**Affiliations:** 1 Molecular Microbiology (Virology), Institute of Medical Microbiology and Hygiene, Universität Regensburg, Regensburg, Germany; 2 Institute of Clinical Microbiology and Hygiene, Universität Regensburg, Regensburg, Germany

## Abstract

The partially protective phenotype observed in HIV-infected long-term-non-progressors is often associated with certain HLA alleles, thus indicating that cytotoxic T lymphocyte (CTL) responses play a crucial role in combating virus replication. However, both the vast variability of HIV and the HLA diversity impose a challenge on elicitation of broad and effective CTL responses. Therefore, we conceived an algorithm for the enrichment of CD8^+^ T cell epitopes in HIV’s Gag protein, respecting functional preservation to enable cross-presentation. Experimentally identified epitopes were compared to a Gag reference sequence. Amino-acid-substitutions (AAS) were assessed for their impact on Gag’s budding-function using a trained classifier that considers structural models and sequence conservation. Experimental assessment of Gag-variants harboring selected AAS demonstrated an apparent classifier-precision of 100%. Compatible epitopes were assigned an immunological score that incorporates features such as conservation or HLA-association in a user-defined weighted manner. Using a genetic algorithm, the epitopes were incorporated in an iterative manner into novel T-cell-epitope-enriched Gag sequences (TeeGag). Computational evaluation showed that these antigen candidates harbor a higher fraction of epitopes with higher score as compared to natural Gag isolates and other artificial antigen designs. Thus, these designer sequences qualify as next-generation antigen candidates for induction of broader CTL responses.

## Introduction

In 2015, 36.7 million people worldwide were infected with HIV^1^. Depending on geographical location, various clades (or subtypes) of the HIV-1 M group are circulating with varying frequencies, with clade C accounting for roughly half of all HIV infections^2^. Within-clade amino acid (aa) diversity is about 12%, and between clades 18%^3^. This enormous diversity is a major hurdle in developing a vaccine against HIV-1.

In general, such a vaccine should elicit both potent humoral and cellular immune responses^4^. Approaches that aim at the elicitation of neutralizing antibodies are directed towards the envelope protein (Env) on the surface of viral particles^5^. In contrast, approaches aimed at eliciting T cell responses are often focused on the group-specific antigen (Gag) that drives the formation of virus particles also referred to as “budding“^6^. The preference for Gag as antigen for T cell vaccines^7^ is due to its abundance in HIV infected cells^8^, its comparably higher conservation (within-clade diversity below 10% on average)^9^, and the association of Gag-specific CD8^+^ cytotoxic T lymphocytes (CTLs) with reduced viral load, or even control, in some HIV-infected patients even without therapy^10–12^.

The specificity of a vaccine is fundamentally determined by the pathogen-derived antigens that are employed. The elicited immune response should be of sufficient breadth, i.e. directed towards multiple determinants, to increase the chance that some of the CTLs induced by vaccination later match the infecting virus. The determinants (epitopes) comprise peptides with a length of about 8 to 12 aa that are presented on MHC class I molecules on the cell surface. Thus, an “optimal” T cell antigen should contain a high number of epitopes that ideally match a large fraction of circulating viruses.

However, considering the high diversity, antigens derived from an arbitrarily selected viral isolate may be quite unrelated to others^13,14^. This problem can be mitigated by using consensus, ancestral, or center-of-tree sequences that aim at minimizing the genetic distance, thus presumably being capable of eliciting broader immune responses^15–17^. Furthermore, the promising concept of so-called mosaic antigens specifically aims at broader CTL responses by enriching potential 9-mer T cell epitopes (PTEs)^18–21^. A limitation is, however, that the PTEs are predicted from HIV sequence analyses as 9-mer aa stretches with certain frequencies in natural isolates. Therefore, they may not represent genuine T cell epitopes, neither are they selected based on favorable immunological features. Other approaches aim at increasing the breadth by using artificially generated antigens that focus CTL responses on conserved parts of HIV proteins, like the HIVconsv^22^ or the p24CE design^23,24^. An alternative concept is to string together selected antigenic determinants flanked by spacers facilitating epitope processing^25–27^. However, such designs may give rise to T cell responses toward neo-epitopes generated by artificial junctions.

Given these limitations, here we describe an algorithm for the generation of full-length Gag variants that are enriched with experimentally identified, genuine CD8^+^ T cell epitopes, thus potentially eliciting broader CTL responses in a vaccination setting. As a prerequisite, these novel Gag variants shall retain their function to drive formation of virus-like particles (VLPs), as this may have a major impact on the immune response. For instance, VLPs might be taken up by professional antigen presenting cells (APCs) for cross-priming. It has been shown that VLPs can be taken up by monocyte-derived dendritic cells in an *ex vivo* assay^28^ and also induce CTL responses in mice^29^. Principally, a VLP resembles the pathogen more closely, and uptake of a VLP would at once deliver several hundred copies of Gag^30^ thus overcoming processing limitations^31^. Indeed we have shown that budding-competent Gag within a larger antigen is more immunogenic in mice than a budding-defective variant^32^. Moreover, DNA and viral vectored vaccine candidates harboring functional Gag antigens were highly immunogenic in non-human primates^33,34^ and clinical trials^35^.

## Results

The aim of this study was to develop an algorithm for the generation of sequences encoding artificial variants of the HIV-1 Gag protein that are enriched with experimentally identified, and thus validated, genuine CD8^+^ T cell epitopes. These should be selected based on a combination of immunological quality parameters in a way that also allows user-defined weighting of these criteria to tailor the sequences toward desired properties. At the same time, epitopes predicted to interfere with Gag’s budding-capacity should be excluded.

The projected algorithm for the generation of T-cell-epitope-enriched Gag (TeeGag) variants follows four basic steps: (i) Mapping: allocation of any given epitopes’ position within a Gag reference sequence and identification of amino-acid substitutions (AAS). (ii) Functional assessment of AAS: employment of a classifier to decide whether an AAS would cause a budding defect. (iii) Epitope scoring: calculation of an immunological quality score from several weighted epitope-specific properties. (iv) Antigen generation: assembly of sets of TeeGags, optimized regarding overall number and score of epitopes.

### Epitope-enrichment algorithm

#### Mapping

Initially all Gag-derived, human CD8^+^ T cell epitopes were retrieved from the Los Alamos National Laboratory (LANL) HIV Molecular Immunology Database. In total, 2688 epitopes were downloaded as raw data. The list of epitopes was then subjected to an initial quality control and was manually curated to remove or correct erroneous and undesired entries, leading to exclusion of 99 epitopes (see Supplementary Dataset 1). From the remaining 2589 epitopes, redundant entries for epitopes with identical sequences were removed, while the annotated parameters were merged. Moreover, annotations of epitopes that are fully included in longer epitopes (referred to as subepitopes, and superepitopes, respectively) were also assigned to the respective superepitope. Thus, a list of 691 unique annotated epitopes was compiled (Supplementary Dataset 2) that constituted the input data for the following steps.

**Figure 1:**
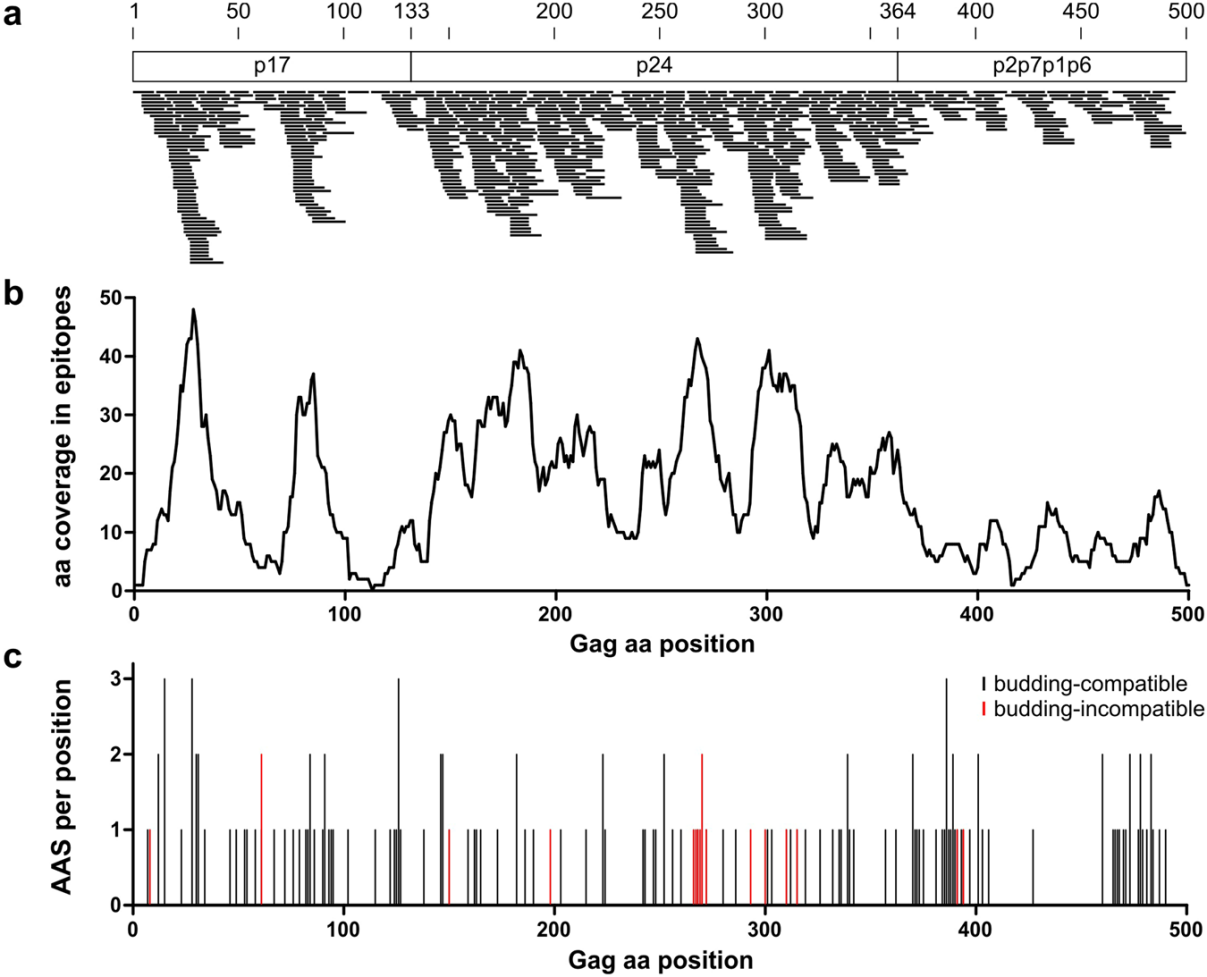
Distribution of epitopes and amino acid substitutions (AAS) across the Gag sequence. (**a**) Location of epitopes within the 500 aa Gag protein that is divided into the three parts p17, p24, and p2p7p1p6 here. Each small black bar represents one epitope with its exact location drawn to scale. (**b**) Number of occurrences of each amino acid position within the epitope set. (**c**) Distribution of AAS per position in Gag as compared to the HXB2 reference sequence. AAS predicted to cause a budding-defect are highlighted in red.

Every epitope was then aligned against the reference sequence HXB2 (Accession-Nr. K03455.1)^36^. The epitope distribution is shown in Fig. 1a and the numbers of epitopes covering each aa position in Gag are shown in Fig. 1b. Within p17, there are two stretches that are part of more than 30 epitopes. p24 exhibits the highest epitope coverage, also with several stretches being present in more than 30 epitopes, while p2p7p1p6 shows the lowest coverage. For each epitope, amino acids differing from the HXB2 reference sequence, referred to as amino acid substitutions (AAS), were determined. A total of 158 AAS were identified, with 380 epitopes harboring no AAS and the remaining 311 epitopes harboring 1.6 AAS on average. Figure 1c shows that the AAS are distributed quite uniformly across the Gag protein affecting 128 positions (25.6%). There are only few positions where an AAS to more than one different amino acid occurred (22 × 2, 4 × 3 different amino acids).

#### Functional assessment

A multi-dimensional classifier was conceived that takes structural and sequence-based properties into account to predict whether an individual AAS is likely to be compatible with, or detrimental to the budding-capacity of Gag. Formation of Gag-VLPs is desired because of potential immunological benefits of particles such as the capacity to be taken up by APCs for cross-presentation.

Four different structure-based discriminatory features were envisaged that are based on the calculation of discrete optimized protein energy values (DOPE)^37^ for each AAS modelled into a structure of the respective Gag-domain using homology-modelling with the MODELLER-tool^38^. For this, HXB2 reference structures for each of the five domains were built initially with MODELLER (see Methods). For p1, no template structure was available, thus one AAS could not be classified. In detail, the four features are (i) the DOPE value at the AAS position (local DOPE), (ii) the DOPE-value-difference between the wildtype and the mutant structure at this position (local ΔDOPE), (iii) the DOPE-value-difference summed up for all aa positions (global ΔDOPE), and (iv) the DOPE-score-differences summed up for a window of twelve amino acids around the AAS position (window ΔDOPE).

For the sequence-based discriminatory feature, a position-specific substitution matrix (PSSM) based on a pre-computed, curated Gag-alignment from the LANL database (2013 filtered web alignment) was built. For each position within Gag, this matrix contains the respective counts of all 20 amino acids occurring in the alignment. As even these 5001 sequences only represent a small subset of all sequences occurring in nature, non-observed substitutions were accounted for by adding artificial pseudo-counts to the PSSM^39^. For an AAS, the score is calculated as logarithm of the normalized frequency of this amino acid in the PSSM.

**Figure 2:**
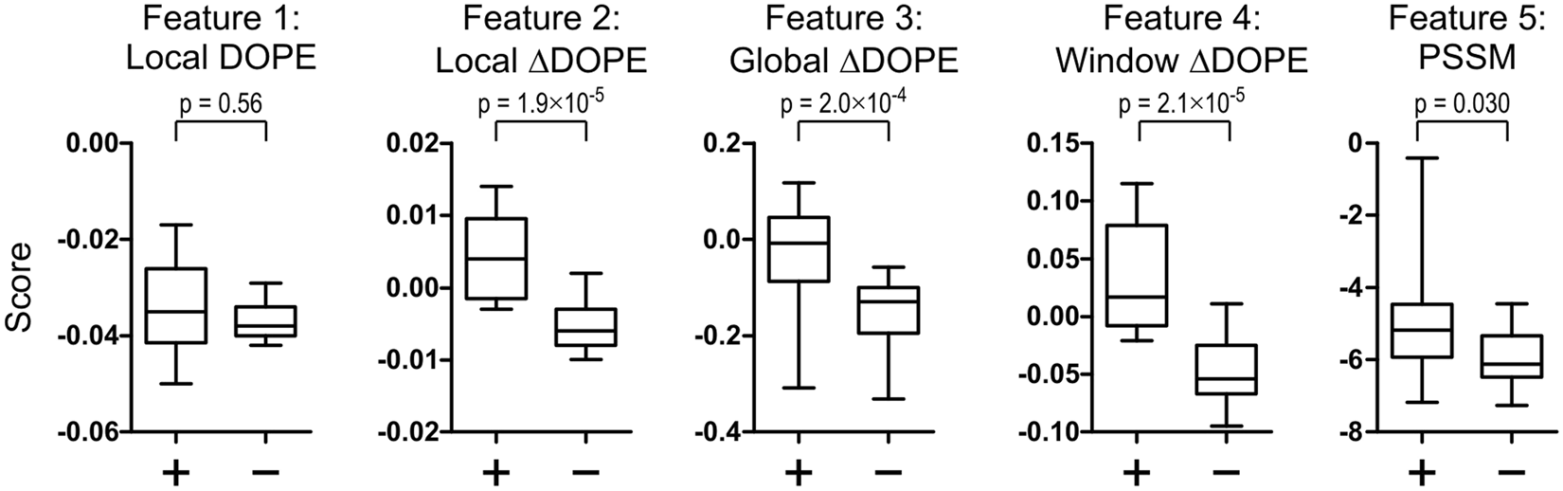
Group-separation of the training-set data for the five discriminatory features. The scores were calculated for all AAS from the training set that were grouped according to their known effect on Gag budding (budding-compatible AAS are indicated by (+), budding-incompatible by (−) on the x axis). Boxes show the median and 50% quartiles, whiskers the 95% percentile. p-values of a Wilcoxon rank sum test are given.

Next, it was assessed how well these five features can discriminate a set of 37 AAS with known effect on budding of Gag (obtained from^40^). Five mutations were left out of the training set as they lie within six aa of the p17 ends, so that structural feature 4 could not be calculated. The scores for each feature were calculated for the 32 AAS and the group separation of the 17 budding-compatible and the 15 budding-incompatible AAS was evaluated. As shown in Fig. 2, all features except for the local DOPE led to a significant group separation (p < 0.05, Wilcoxon rank sum test) with local ΔDOPE exhibiting the best discriminatory capacity. However, there was still substantial overlap between the groups, so that it was decided to improve the prediction by using feature combinations.

To assess such combinations, the easy to implement Fisher’s linear discriminant analysis was employed^41^. All 31 possible combinations were naively tested with 10-fold cross-validation for best discrimination of the AAS in the training-set (Table 1). The highest accuracy, i.e. best classification of AAS as true positive (budding-compatibility) and true negative (budding-defect), was obtained with the combination of the global ΔDOPE-score and the sequence-conservation, achieving 86%. With this feature combination, the algorithm had a precision of 99%, while the negative predictive value (NPV) was 78% at most. This implicates a higher prediction quota of true budding-positive AAS, whereas the predicted budding-negative AAS would include more false negatives. Accordingly, the specificity peaked at 99%, while the sensitivity was 75%.

**Table 1:** Test characteristics for all combinations of the five discriminatory features when classifying the training set data.

Feature combination	Accuracy	Precision (PPV)	NPV	Sensitivity	Specificity	MCC
3 + 5	86 ± 3	99 ± 3	78 ± 4	75 ± 6	99 ± 3	0.75 ± 0.06
1 + 3 + 5	84 ± 4	98 ± 4	75 ± 4	71 ± 6	98 ± 4	0.71 ± 0.07
4	81 ± 2	86 ± 1	76 ± 3	75 ± 4	87 ± 0	0.62 ± 0.03
4 + 5	80 ± 3	86 ± 1	75 ± 4	74 ± 5	87 ± 0	0.61 ± 0.05
1 + 3	79 ± 3	83 ± 4	75 ± 2	77 ± 1	82 ± 5	0.58 ± 0.06
3 + 4	79 ± 2	86 ± 1	73 ± 3	72 ± 4	87 ± 1	0.59 ± 0.04
3	78 ± 4	80 ± 5	77 ± 3	80 ± 3	76 ± 7	0.57 ± 0.08
2	78 ± 1	86 ± 0	72 ± 1	71 ± 1	87 ± 0	0.58 ± 0.01
2 + 4	78 ± 1	86 ± 0	72 ± 2	71 ± 2	87 ± 0	0.58 ± 0.02
2 + 5	78 ± 1	86 ± 0	72 ± 2	70 ± 2	87 ± 0	0.57 ± 0.02
3 + 4 + 5	77 ± 3	85 ± 1	72 ± 3	69 ± 5	87 ± 0	0.56 ± 0.05
2 + 4 + 5	77 ± 2	85 ± 1	71 ± 3	69 ± 4	87 ± 0	0.56 ± 0.04
2 + 3	75 ± 1	85 ± 2	69 ± 1	65 ± 2	87 ± 1	0.53 ± 0.02
2 + 3 + 4	75 ± 1	85 ± 1	69 ± 1	65 ± 2	87 ± 1	0.53 ± 0.03
1 + 4 + 5	75 ± 3	85 ± 1	69 ± 3	65 ± 5	87 ± 0	0.52 ± 0.05
2 + 3 + 5	75 ± 2	85 ± 1	68 ± 2	65 ± 3	87 ± 1	0.52 ± 0.03
2 + 3 + 4 + 5	74 ± 2	84 ± 1	68 ± 2	63 ± 4	87 ± 1	0.51 ± 0.04
1 + 4	74 ± 2	84 ± 1	67 ± 3	63 ± 4	87 ± 0	0.50 ± 0.04
1 + 2 + 5	73 ± 2	84 ± 1	66 ± 2	61 ± 3	87 ± 0	0.49 ± 0.03
1 + 2	73 ± 2	84 ± 1	66 ± 2	61 ± 3	87 ± 0	0.49 ± 0.03
1 + 2 + 4	73 ± 2	84 ± 1	66 ± 2	61 ± 4	87 ± 0	0.49 ± 0.03
1 + 2 + 4 + 5	73 ± 2	84 ± 1	66 ± 3	60 ± 5	87 ± 0	0.48 ± 0.04
1 + 3 + 4 + 5	73 ± 3	83 ± 1	66 ± 3	60 ± 6	87 ± 1	0.48 ± 0.05
1 + 3 + 4	72 ± 3	83 ± 1	65 ± 3	59 ± 5	87 ± 0	0.47 ± 0.04
5	72 ± 2	75 ± 2	68 ± 2	69 ± 2	74 ± 3	0.44 ± 0.04
1 + 2 + 3 + 5	71 ± 2	83 ± 1	64 ± 2	57 ± 4	87 ± 0	0.45 ± 0.03
1 + 2 + 3	71 ± 2	83 ± 1	64 ± 2	57 ± 4	87 ± 1	0.45 ± 0.04
1 + 2 + 3 + 4 + 5	71 ± 2	83 ± 1	64 ± 2	56 ± 4	87 ± 0	0.45 ± 0.04
1 + 2 + 3 + 4	70 ± 2	83 ± 1	64 ± 2	56 ± 5	87 ± 0	0.44 ± 0.04
1 + 5	68 ± 3	73 ± 2	64 ± 4	63 ± 6	73 ± 2	0.36 ± 0.06
1	58 ± 2	64 ± 3	55 ± 2	51 ± 4	67 ± 3	0.18 ± 0.05

All numbers are percent values. PPV = Positive predictive value, NPV = negative predictive value; table sorted from highest to lowest accuracy. MCC = Matthews correlation coefficient.

As preservation of budding-competence was considered mandatory for the final Gag variants, the feature-combination with the highest precision was chosen. Thus, the likelihood of classifying a budding-incompatible AAS falsely as budding-compatible is minimized, while accepting a slightly higher rate of falsely classifying a truly budding-compatible AAS as incompatible. Therefore, the classifier algorithm was set to rate each AAS based on the combination of features 3 and 5, and was eventually trained with the full training set.

The classifier then identified 18 of 158 (11%) AAS to be budding-incompatible, leading to the exclusion of 21 epitopes (3%) harboring at least one of these detrimental AAS. As shown in Fig. 1c, most of them (72%) are located within p24 which is in line with the high conservation of this part of Gag^42^. In addition, an analysis of all possible mutations for each position in Gag was performed (except p1; 484 × 19 = 9196 theoretically possible AAS). Here, 4839 out of 9196 (52.6%) were predicted to be budding-incompatible. This shows that – as expected – experimentally identified epitopes are significantly depleted of budding-incompatible AAS (p < 2.2×10^−16^, Fisher’s exact test).

#### Epitope Scoring

The set of 670 epitopes that were predicted to be free of budding-incompatible AAS was used further. As the epitopes differ in their immunological and virological characteristics, epitopes should also be selected based on such properties, thus conditioning the probability of an epitope’s incorporation into the finally assembled antigen sequence. For this, a scoring scheme based on the annotation data that is available in the LANL database was conceived. The following parameters were considered for each epitope: (i) HLA-association, (ii) subtype of the parental virus, (iii) response in long-term non-progressors (LTNPs), (iv) experimentally determined recognition rate, (v) conservation.

As HLA alleles are not uniformly distributed, it might be beneficial to preferentially incorporate epitopes that are restricted by more common HLAs. The HLA restriction reported for the epitopes in the LANL database is, however, quite heterogeneous. For instance, the HLA association is only given for 59.5% of the epitopes. By assigning the HLA-association of sub-epitopes also to their corresponding super-epitopes and using only allele groups, this is harmonized to a limited degree. The HLA-association-score is then calculated by summing up the global HLA population frequencies^43^. It is important to emphasize that the scores are most likely biased because not all epitopes have been characterized to the same degree and with comparable methods.

It might be relevant for some applications to adapt a potential antigen to one or several clades that for example are matched to viruses circulating in the target population. Thus, a parameter relating each epitope to the virus subtype from which it is derived was included. This information is again part of the LANL-metadata for each epitope. By assigning individual weights for each clade, the relative contribution can be defined by the user. The subtype-score is then calculated for each epitope by summing up the weights assigned to the clades with which the epitope is associated. Finally, the overall score gets normalized. For the proof-of-concept, we assigned weights that represent the relative world-wide frequency of each clade^44^.

The boolean LTNP parameter receives a value of 1 if a T cell response against this epitope has been found in LTNPs or elite-controllers according to the LANL metadata.

For some epitopes it has been investigated how many people in a study population do have a T cell response against the respective peptide. If this information was provided among the metadata of the epitope’s database entry, this fraction was used as the “experimental recognition” score.

Finally, to assign a measure of conservation to each epitope, it was assessed how frequently the exact epitope sequence occurs in the filtered web-alignment of full-length HIV-1 Gag sequences. As the fraction of sequences from a specific subtype in the alignment does not correlate with the natural subtype frequency (especially over-representation of subtype B), the epitope frequency was assessed for each subtype individually, weighted according to the natural subtype distribution, summed up for all subtypes and finally normalized.

Eventually, the overall epitope score was then calculated by summing up the five weighted individual scores. The weight (w) can be user-defined. For the proof-of-concept, the following values were used: w(HLA-association) = 6; w(subtype) = 3; w(LTNP) = 6; w(experimental recognition) = 1; w(conserved) = 3.

#### Antigen Generation

Finally, a genetic algorithm was employed to assemble artificial T-cell-epitope-enriched Gag variants with the maximal overall score by including the highest-possible number of highest-scoring epitopes. Overlapping epitopes can be combined if the overlapping part has identical aa sequence. Here, the genetic algorithm is important for approximating the best solution for combinations of such compatible epitopes. Finally, unassigned aa positions were filled with the respective aa from HXB2 to come up with a full-length Gag protein.

**Figure 3:**
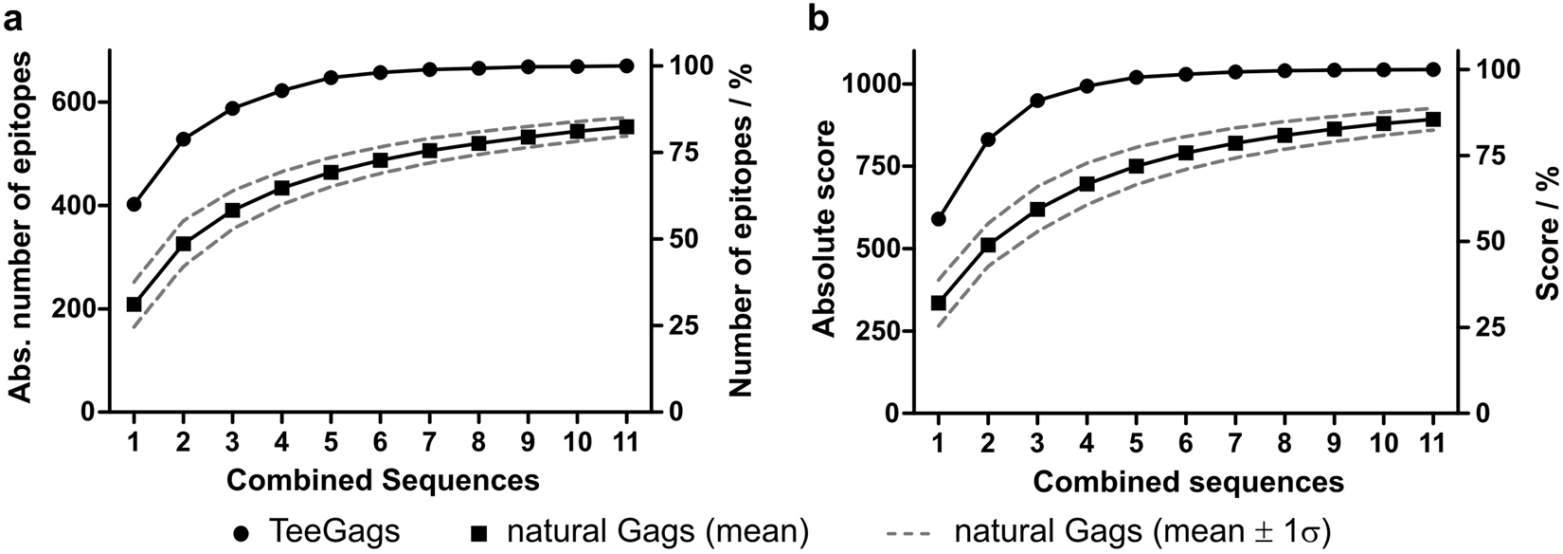
Progress of epitope coverage and total score for combinations of complementary TeeGags and random combinations of natural sequences. The overall number of different epitopes (**a**) and the overall score (**b**) of a set of iteratively generated TeeGags were determined and are given for round 1 and cumulatively summed up from rounds 1 to the number indicated on the x-axis, in absolute numbers (left y-axis) and in percent (right y-axis). For comparison, the corresponding values are given for all 5001 natural Gag sequences (round 1) or 100,000 combinations of 2 to 11 randomly selected natural sequences as mean with 1-σ-interval.

The genetic algorithm was executed in iterations. Epitopes integrated into the first Gag variant were removed from the input data set for the subsequent assembly. By this, all 670 epitopes could be integrated into a set of 11 TeeGags. The overall number of epitopes, as well as the cumulative score of combined complementary TeeGags, rapidly increased and soon reached a plateau (Fig. 3). Whereas the sequence of the Gag variant from the first iteration covers 60% of the epitopes and 56% of the score, the set of the first three TeeGags combined already covers about 88% of all epitopes representing about 91% of the overall score. In contrast, random combinations of three natural isolates cover on average only 58% of all epitopes and 59% of the score. For the following analyses we generated a prototype set of three such Gag variants, termed TeeGag1, TeeGag2, and TeeGag3 (sequences in Supplementary Dataset 3). Compared to the HXB2 wildtype Gag sequence, these variants harbor 28, 39, and 40 AAS, respectively (alignment in Supplementary Fig. S1). This leads to inclusion of an additional 58, 102, and 53 epitopes into TeeGag1 to TeeGag3, that are not included in HXB2 or the previous TeeGag iteration, respectively.

### Experimental Antigen Validation – Budding and release of VLPs from cells

Next, the performance of the classifier regarding the correct prediction whether inclusion of an AAS into the Gag reference protein causes a budding-defect was experimentally assessed. For this, the influence of each AAS contained in either TeeGag1, TeeGag2, or TeeGag3 on VLP-formation was analyzed. Plasmids carrying the codon-optimized HXB2 *gag* gene with a single one of the 84 AAS were constructed. As reference, another 18 plasmids with AAS predicted to cause a budding-defect were generated.

**Figure 4:**
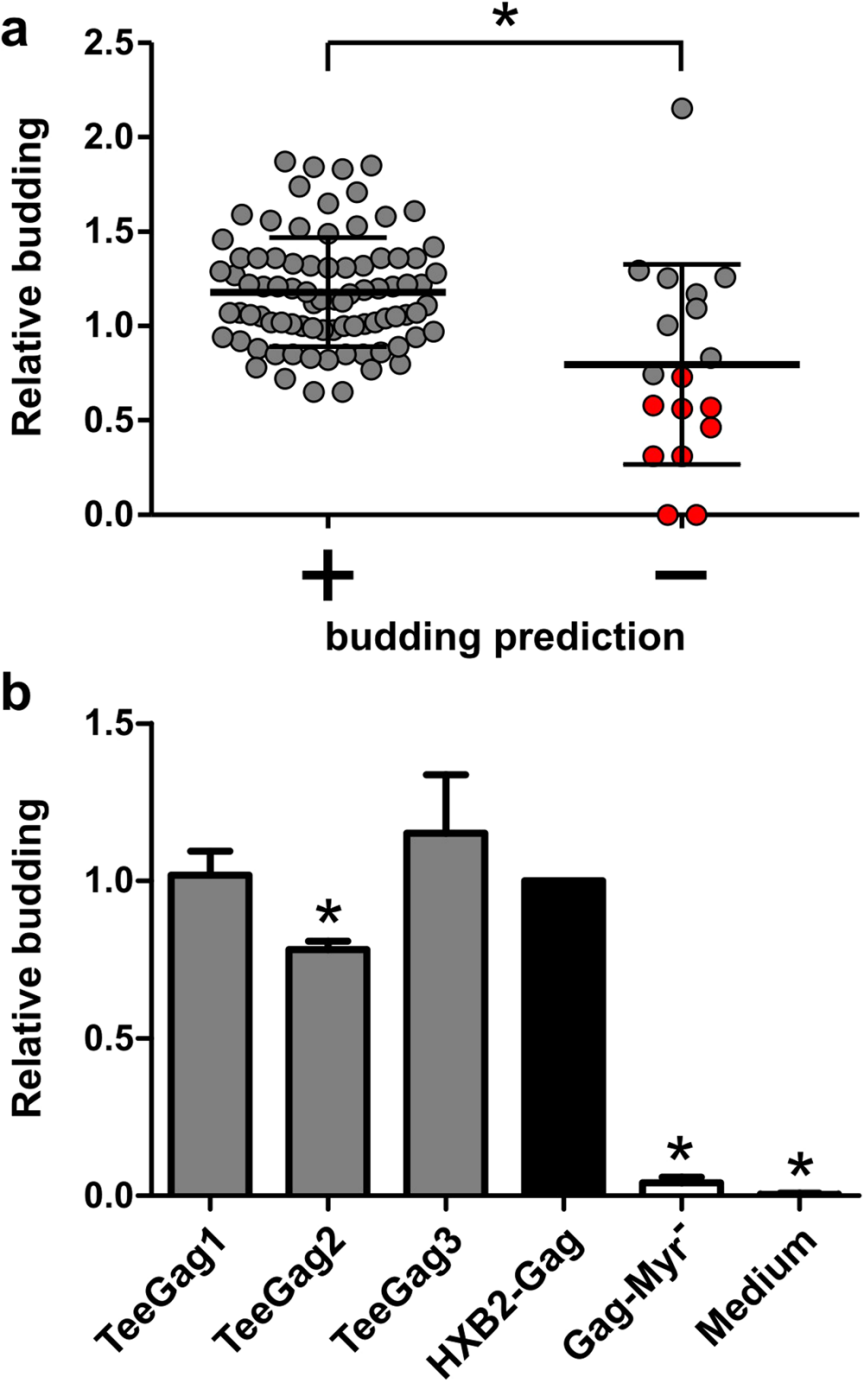
Budding-capacity of the Gag-variants. (**a**) HEK293T cells were transfected with plasmids encoding HXB2-Gag-wildtype or Gag with individual AAS, predicted to be budding-compatible (+) or not (−). A SEAP-plasmid was co-transfected to allow normalization of variations in transfection efficiency. 48 h later, the supernatants were harvested and assayed for Gag-content using a p24-ELISA, and for SEAP-content using a colorimetric SEAP-activity-assay. Relative budding is expressed as the p24/SEAP ratios of the respective Gag-variant relative to the ratio of the wildtype-Gag sample. Individual values are shown, representing the mean of duplicate p24/SEAP-measurements of three independent transfections per variant. Variants that exhibit a significant reduction of relative budding in a t-test (p < 0.05, with Bonferroni-correction) are highlighted in red. In addition, the mean and standard deviation are shown for the groups; the asterisk denotes a significant difference between the groups in a t-test (p < 0.05). (**b**) HEK293T cells were transfected with plasmids encoding His-tagged versions of the three TeeGag-variants, HXB2-Gag, or the myristoylation-defective Gag-variant (Gag-Myr^−^). Supernatants were harvested after 48 h. Equal volumes of dilution-series were applied to a nitrocellulose-membrane in a slot-blot. His-tagged Gag was detected via ECL using a biotinylated His-tag-specific-antibody and a streptavidin-peroxidase-conjugate. Relative budding is expressed as the ratio of the SEAP-normalized densitometrically measured amounts of the respective variants compared to HXB2-Gag. Asterisks denote significant differences to HXB2-Gag in a t-test (p < 0.05).

All plasmids were transfected in parallel into HEK293T cells and supernatants were analyzed 48 h later. As shown in Fig. 4a, all variants predicted to be budding-competent indeed exhibited Gag release. Compared to the wildtype HXB2 Gag, some variants showed slightly decreased or increased particle production, yet no variant was significantly impaired. In contrast, among the variants predicted to have a budding-defect, 9 exhibited a significant reduction in Gag release. In total, the groups of Gag variants predicted to be budding-competent or not, were significantly different, thus confirming the discriminatory power of the classifier.

In addition, plasmids encoding the three TeeGags were generated, and, as negative control, a myristoylation-deficient HXB2-Gag variant. To rule out that an AAS affects recognition by Gag-specific antibodies, a His-tag was added for detection of the proteins. All three TeeGag variants did form particles despite the inclusion of up to 40 point mutations (Fig. 4b). However, particle release of TeeGag2 was slightly, but significantly, impaired.

Comparing the experimental results with the theoretical predictions by the classifier shows that apparently a precision (positive predictive value) of 100% was obtained. As described above, the highest value of this analytical parameter guided selection of the feature-combination for the classifier, and achieved 98% based on the training-set-data. The negative predictive value was only 50%, reflecting that false exclusion of some AAS is accepted in favor of not including detrimental AAS. The sensitivity and specificity were 90% and 100%, respectively.

### Computational Antigen-Validation

A computational analysis was performed to validate the newly designed TeeGag variants regarding the absolute numbers of epitopes contained and their overall score. We compared these parameters for the TeeGags, natural Gag-isolates, as well as the artificial consensus (con), ancestral (anc), center-of-tree (cot), and mosaic-Gag (mos) designs that all represent full-length Gag-proteins.

**Figure 5:**
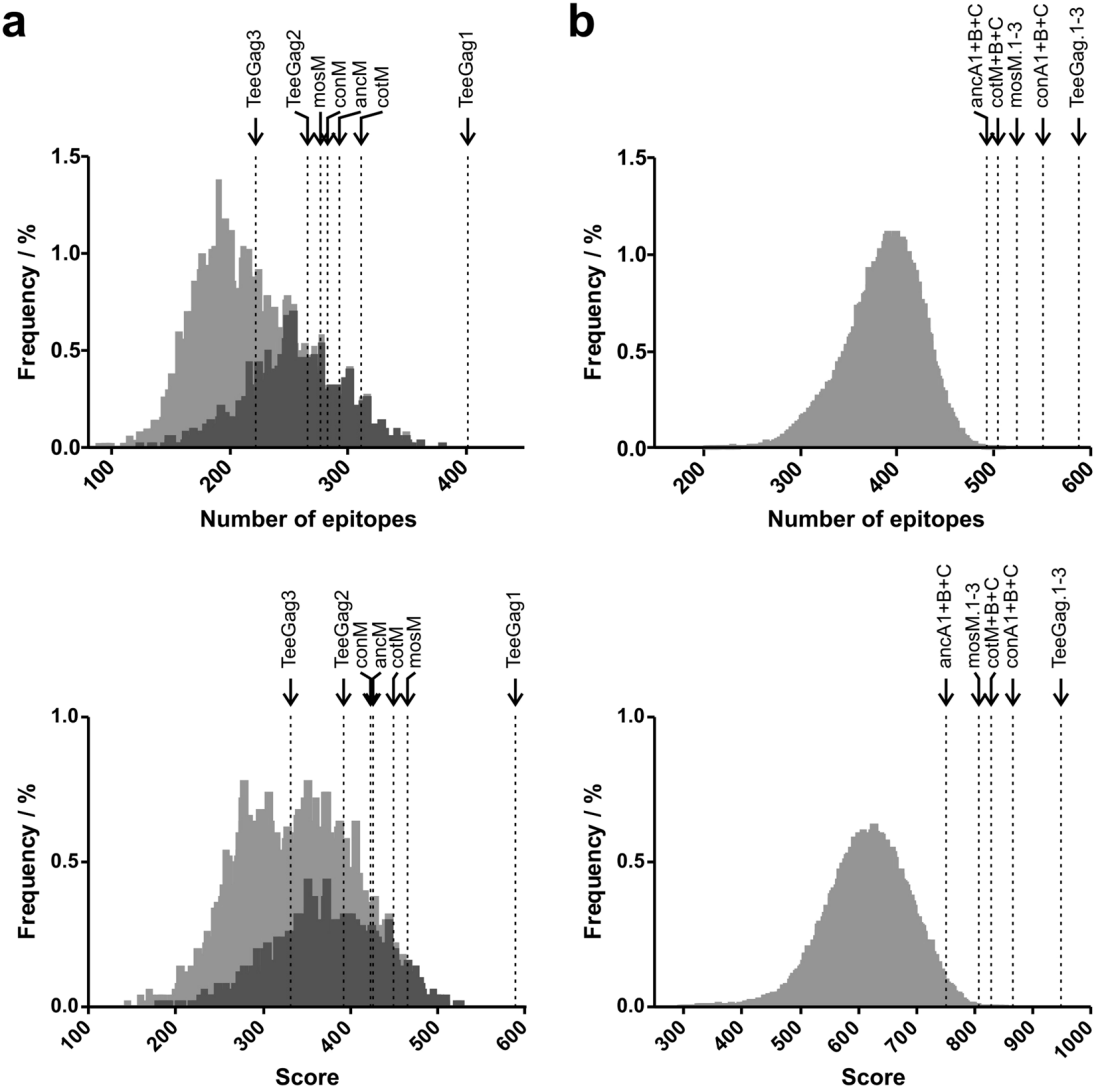
Comparison of epitope coverage and score of natural Gag-sequences and artificial designs. (**a**) Frequency of the 5001 monovalent natural sequences (shown in grey, with B-clade-sequences shaded in dark grey) harboring the respective numbers of epitopes (top panel) or with the respective scores (bottom panel) as indicated on the x axis. The epitope numbers and scores of the various artificial Gag-designs (consensus sequence = con, ancestral = anc, center-of-tree = cot, mosaic = mos; followed by M referring to the whole group M of HIV-1, or A1, B, or C, referring to the respective clades) are highlighted by vertical arrows and dashed lines positioned according to the corresponding values. (**b**) Frequencies of 100,000 trivalent, randomly combined natural sequences (grey), as well as of trivalent Gag designs as indicated (arrows and dashed lines).

As shown in Fig. 5, natural isolates on average cover 208 epitopes (25.4% of the score), with the best natural isolate HXB2 covering 380 (50% score). As highlighted in the figure, natural B clade isolates score higher which is due to B-clade-derived epitopes being overrepresented in the database.

The quality of natural isolates is surpassed by TeeGag1 with 402 epitopes (56.5% score). The M group consensus (conM) has a coverage of 283 epitopes (40.6% score), the ancestor (ancM) of 293 (40.9% score), the center-of-tree (cotM) of 311 (43.1% score), and the mosaic (mosM) 277 (44.7% score). Values for coverage and scores relating to the whole epitope set for artificial Gag sequences designed toward individual clades are given in Supplementary Table S1.

As a large fraction of the higher-scoring epitopes are contained in TeeGag1 and thus are excluded from the subsequent complementary TeeGags, the epitope coverage is lower in TeeGag2 and TeeGag3, with 266 epitopes (37.6% score) and 222 epitopes (31.8% score), respectively. The advantage of using complementary antigen sets becomes evident when the quality of trivalent antigen-sets is compared. Here, the combination of TeeGag1, TeeGag2, and TeeGag3 covers 588 epitopes with 91.0% of the score. The combination of mosaics mosM.1, mosM.2, and mosM.3 which similarly consist of complementary sequences, achieves a coverage of 524 epitopes (77.4% score). By virtue of their design, complementary con and anc sequences do not exist. However, combining the sequences of the three most dominant clades (C, B, A1) leads to a coverage of 551 epitopes (83.1% score), and 493 epitopes (72.0% score) for consensus and ancestral sequences, respectively. Taken together, although all these alternative sequence designs reach substantial theoretical coverage in this computational analysis, the TeeGags are superior in relation to the analyzed epitope set.

In addition to TeeGags 1 to 3, we also generated sets of clade-specific TeeGags for the most frequent clades, i.e. TeeGagC, TeeGagA, and TeeGagB (see Dataset S3) that are generated by the same algorithm but from reduced sets of epitopes that include only determinants specific to the respective clade. Thus, these sequences are useful for approaches where antigens are desired that match a certain clade. Importantly, due to the curation of the epitopes to be used, these designs are not prone to biases resulting from overrepresentations of epitopes derived from certain clades in the database, as is currently the case for subtype B.

Similar to the M-group-specific TeeGags described above, these designs reach substantial coverage both for the number of included epitopes as well as the associated scores (Supplementary Fig. S2). For instance, TeeGagC1 contains 150 of 204 C-clade-associated epitopes covering 78% of the immunological score, and the trivalent TeeGagC.1-3 even contains 189 epitopes (96% score).

**Figure 6:**
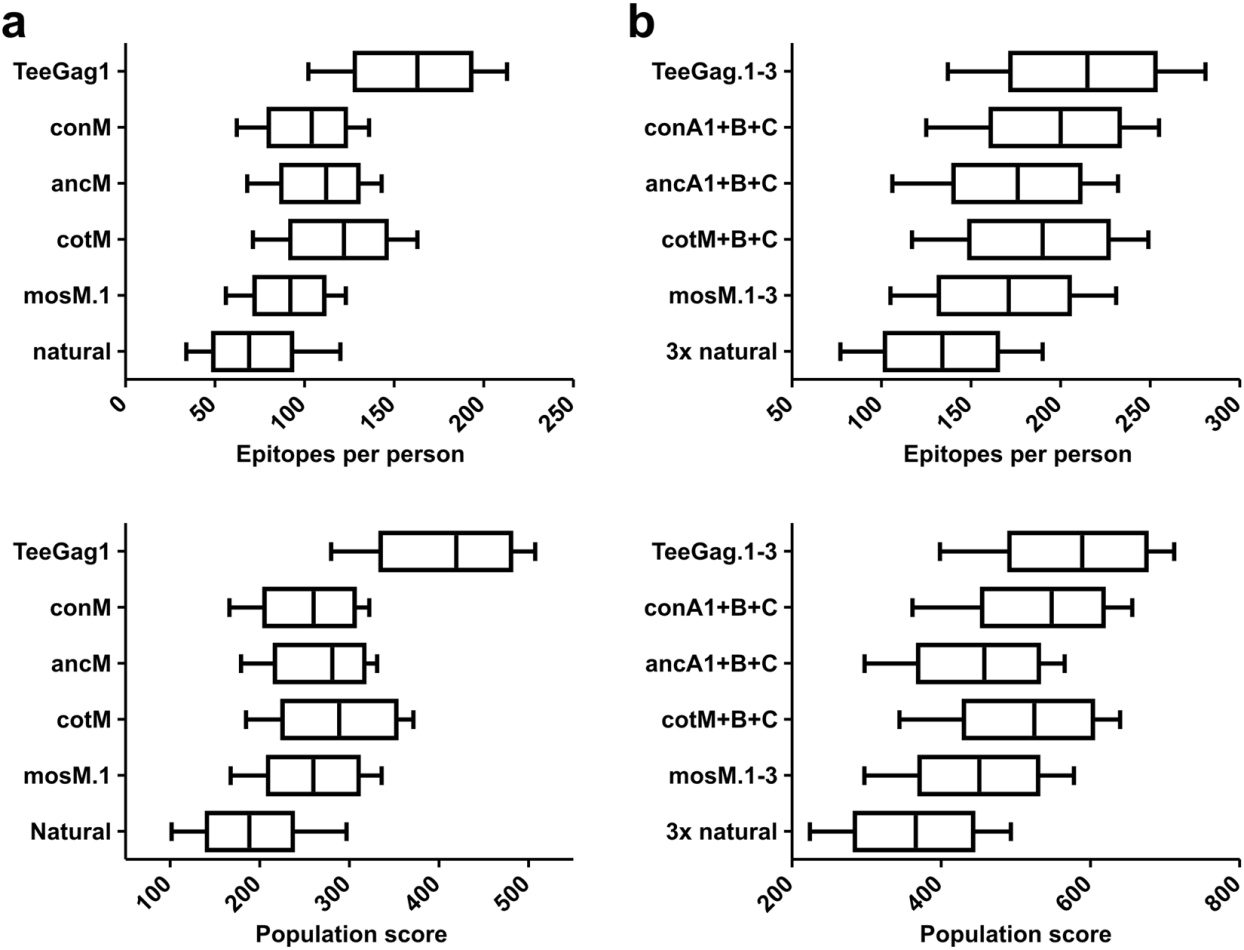
Population-coverage of various Gag-designs and natural Gag-sequences. The number of epitopes (top panels), and the overall scores (population-score, lower panels) for (**a**) monovalent, or (**b**) trivalent Gags as indicated on the y axes, was determined for 1000 HLA haplotypes that were randomly generated by selecting two HLA-A, -B, and -C alleles each, respecting the allele frequencies. Boxes show the median and 50% quartiles, whiskers the 10 and 90% percentiles.

As detailed above, the HLA-association of epitopes was factored into the TeeGag-design. To get an indication of how many epitopes fitting to a persons’ HLAs are potentially present in different Gag-designs, a population with representative haplotypes was simulated and the number of epitopes that are restricted by at least one HLA-molecule was determined (Fig. 6a). As additional measure, a population-score was determined by summing up the individual scores of the fitting epitopes. TeeGag1 contained a median of 163 epitopes (with median population-score of 419), whereas natural isolates had a median of only 69 epitopes, and the other designs ranged from 92 for mosM.1 to 122 epitopes for cotM. Similarly, trivalent combinations of TeeGags are superior both regarding epitope number (median 215) and population score (median 589), although the difference to the other artificial designs is smaller (Fig. 6b). Trivalent combinations of randomly picked natural sequences are clearly inferior. Additional data is given in Supplementary Fig. S3.

**Figure 7:**
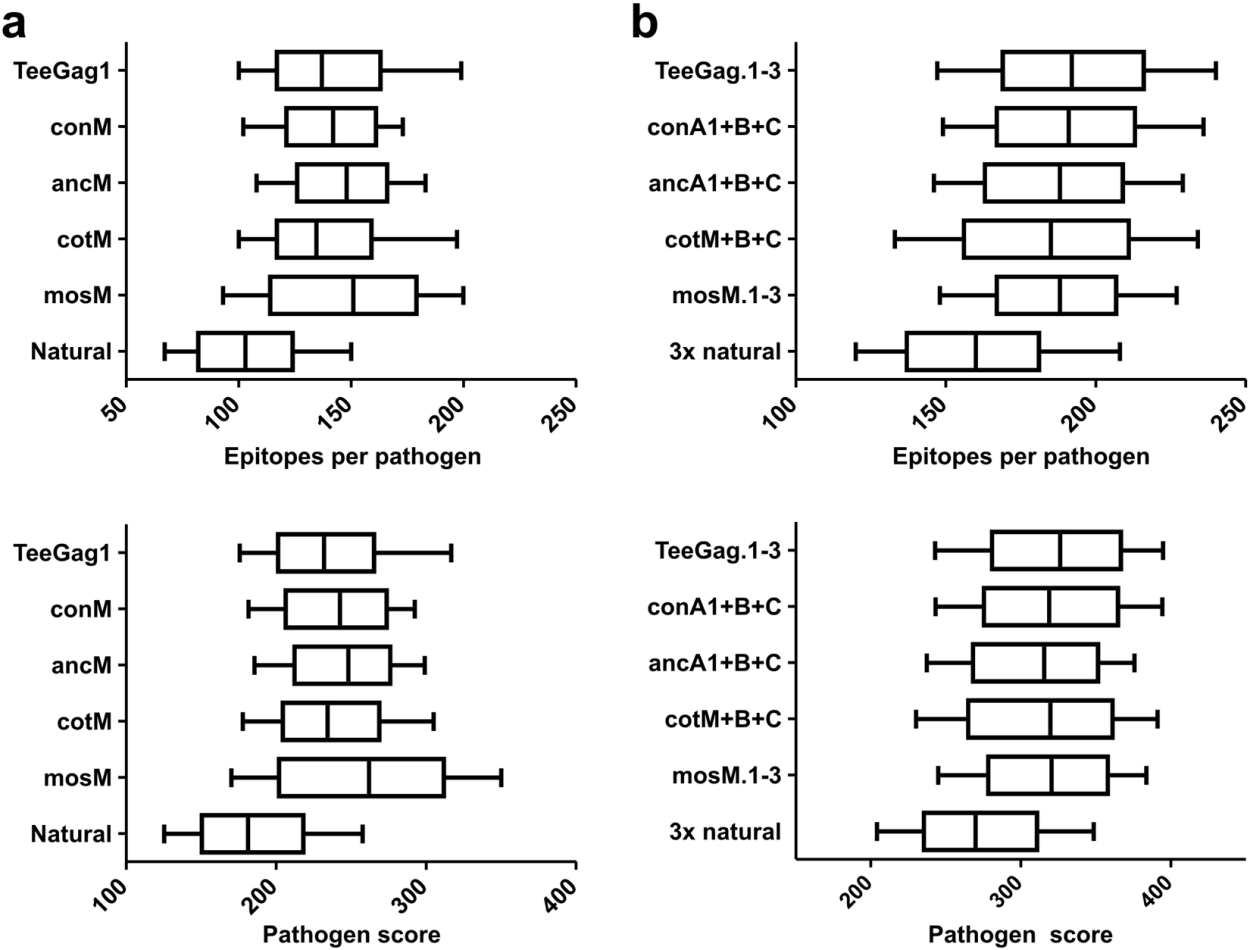
Pathogen-coverage of various Gag-designs and natural Gag-sequences. The number of epitopes (top panels), and their summed up scores (pathogen-score, lower panels) for (**a**) monovalent, or (**b**) trivalent Gags as indicated on the y-axes, was determined for 1000 randomly picked natural Gag-isolates (pathogens), respecting the clade-frequencies. Boxes show the median and 50% quartiles, whiskers the 10 and 90% percentiles.

Analogously, the clades from which the epitopes originate had been factored into the TeeGag-design taking the world-wide clade-frequencies into account. To estimate how many TeeGag epitopes are also contained in sequences of different circulating HIV isolates, 1000 representative natural Gag-sequences (“pathogens”) were randomly selected respecting world-wide clade-frequencies. Then, the number of epitopes present in the respective Gag-antigen and the pathogen at the same time and their combined pathogen-score were determined (Fig. 7a). TeeGag1 contained a median of 137 epitopes (with median pathogen-score of 232), while the natural isolates were again clearly inferior. The other designs were similar, ranging from 135 (cotM) to 151 epitopes (mosM.1). Trivalent combinations of all the designer-Gags were very similar and superior to trivalent, randomly chosen natural Gags (Fig. 7b). Additional data is given in Supplementary Fig. S4.

## Discussion

An efficacious HIV-1 vaccine will have to elicit broadly reactive T cell and antibody responses to address the variability of circulating viruses^13,16^. Gag is the prime antigen for approaches aimed at eliciting CTL responses^7^. Such responses have the capacity to reduce viremia after infection, especially if the response broadly targets several Gag peptides^45^. Moreover, there is an inverse relationship between the breadth of Gag-specific responses and the set-point viral load^46^.

Here, we describe a novel algorithm for the design of T-cell-epitope-enriched Gag antigens (TeeGags) that harbor more and, according to several criteria, better epitopes than arbitrarily selected natural Gag sequences. Compared to the design approach for mosaic antigens^18–20^, there are two major differences: First, the TeeGag-algorithm incorporates epitopes that have been experimentally identified in humans. Second, the TeeGag-algorithm evaluates all epitopes for compatibility with Gag’s budding-function.

Mosaic antigens are generated by enriching PTEs^18,47^. This leads to a very good coverage of highly conserved sequences including T cell epitopes, but less frequent ones or those with different length may be missed. In contrast, the TeeGag-algorithm considers all epitopes in the pre-specified set, in this case all the entries from the LANL database. Yet, a number of relevant epitopes is of course expected to be missed, simply because they have so far not been experimentally identified. A bias of the database entries is for instance evident by the strong overrepresentation of epitopes derived from B clade viruses (see Fig. 5a) that account for only about 10% of the world-wide circulating strains^2^.

Common to both algorithms is that the desired epitopes are present with their defined sequence in the final antigen. However, most likely not all of them will end up as T cell epitopes bound to an MHC I molecule on the surface of a cell as the process of generating peptides for presentation on the cell-surface is highly complex. Each step is prone to a selectivity filter which in sum precludes the formation of many conceivable T cell epitopes, and favors the presentation of others. Although big advances have been made in predicting the outcome of this process^48^, the accurate prognosis of the eventually MHC-I-presented epitopes is still limited^49^. Recently, mass-spectrometric approaches have been developed that can be used to identify peptides eluted from MHC I molecules^50^. However, the method is not yet sensitive enough to pick up the majority of epitopes, currently yielding only 0.5–3% of peptides^51^. Alternatively, individual pMHC-complexes can be detected by mixed-lymphocyte reactions with well-characterized T-cell-lines. However, as only few of them are available a comprehensive analysis is not possible. Additional factors may also influence the final presentation, such as protein processing by the so-called immunoproteasome upon IFN-γ-stimulation^52^.

Preclinical evaluation in animal models will be necessary to further develop TeeGags as antigens. Analyses in mice or macaques will be informative regarding the safety of the antigens and in determining optimal vaccination regimens. However, such models would only yield very limited information on whether the epitope-enrichment of TeeGag’s does lead to broader CTL responses mainly due to the non-human specifics of peptide processing and presentation. For mosaics, it has been shown in mice and macaques that broader T cell responses get primed as compared to antigens from natural isolates^19,20^, but the design-algorithm enriches any frequent 9-mer peptide without considering an association with mouse, macaque, or human MHC molecules so that mosaic antigens are improved irrespective of species. Similarly, conserved element (CE) antigens have been shown to elicit broader responses to the CE-antigens in animal models as compared to full-length antigens^24^, but here as well, the antigens are not specifically tailored toward peptides that are presented on HLA molecules but any reactivity in a CE region generally indicates immunogenicity of the respective CE element. Thus, to assess whether the novel TeeGag antigens can elicit broader CTL responses, it will be necessary to either test their stimulatory capacity with a large panel of T cells from HIV-infected people, or even to assess the breadth of CTL responses that get primed in humans in a clinical trial.

An interesting alternative would be to transfer the concept to SIV antigens that could be evaluated in macaques, as for instance has been successfully demonstrated for conserved elements with a p27Gag-CE design^53^. However, as there are at the moment only few SIV-Gag-derived CTL epitopes identified in macaques in the LANL database, only a limited enrichment of epitopes in an SIV-TeeGag would currently be possible. However, once more epitope sequences become available, this might be a viable model to test whether epitope enrichment translates into broader T cell responses.

As mentioned above, the overall efficacy of a CTL response correlates with the breadth^46^, i.e. the number of different epitopes that are targeted by T cells. However, most likely not all reactivities contribute equally to the overall response. Especially CTLs that are directed toward variable epitopes prone to rapid escape may have only little and transient impact on controlling HIV replication. T cell responses to such epitopes may be of high strength and impede priming of CTL responses toward other epitopes in a phenomenon called immunodominance. To avoid this, strategies have been proposed where the variable regions eliciting such responses are deleted, thus coming up with artificial polypeptides such as p24CE^24^ or HIVconsv^22^. Though these antigens lead to a focusing of the immune response on conserved regions that mostly elicit subdominant responses, the benefits of using the full-length, functional protein are of course lost. Moreover, the unnatural linking of sequences in the artificial antigens may cause further problems^54^, like the generation of neo-epitopes^55^, which is not the case for the full-length Gag designs (i.e. mosaics and TeeGags). Whereas a full-length Gag protein elicited responses of similar breadth but of higher magnitude than the HIVconsv antigen^56^, for p24CE, in contrast, it could be shown that it elicits broader responses recognizing more CE elements than when a full-length Gag was used for vaccination in mice^24^. Similarly, use of an analogous design for SIV, i.e. p27CE, in macaques led to robust CTL responses targeting subdominant epitopes of the conserved elements, thus altering the immunodominance hierarchy^53^. Similar results were obtained for HIV-Env-derived CE-antigens in macaques^57^. Even in the setting of a therapeutic vaccination in SIV-infected macaques, the p27CE was able to elicit focused responses toward the conserved element sequences^58^.

Interestingly, co-delivery of a full-length SIV-Gag together with the p27CE antigen to boost responses initially primed by p27CE proved to be superior to a p27CE-only boost in increasing the magnitude of the response while the breadth was not negatively affected^53^. Thus, even in the context of strategies aimed at focusing of immune responses to conserved regions, TeeGags might be useful as boosting agents as well. Alternatively, the TeeGag-algorithm could be modified to penalize epitopes known to elicit immunodominant responses to prevent their incorporation into the protein.

The other important design feature of the TeeGag-algorithm is the prediction whether an AAS is compatible with VLP formation, as this may be of high importance for priming a Gag-specific CTL response^17^. Put simply, a virus-like structure is what our immune system has learnt to deal with and therefore it is reasonable to assume that a VLP constitutes the best form for delivery of Gag as an antigen.

Therefore, the algorithm encompasses a classifier that judges whether an AAS is likely to be compatible with budding or not. This classifier excellently ruled out budding-incompatible AAS. Interestingly, the fraction of AAS that would cause a budding defect was extraordinarily higher when all theoretically possible AAS were considered. This is in line with an analysis showing that especially p24 is genetically extremely fragile in that it tolerates only very few substitutions^59^. An accurate assessment of the AASs is of prime importance when considering the realization probability of functional Gag-variants with many AASs combined. For example, if the prediction had a precision of only 98%, then a variant like TeeGag1 with 28 mutations would have a realization probability of just 57%, and TeeGag3 with 40 mutations only 45%. Yet, the experimental assessment of the three complementary TeeGags showed that they do form VLPs, although TeeGag2 may be slightly impaired (Fig. 4b). Of course, as the AAS are derived from experimentally identified epitope sequences, one would expect that they were initially derived from replicating viruses. Yet, analysis of the few AAS predicted to be budding-incompatible confirmed that about half of them were truly budding-defective ones. Thus, some epitopes may have been derived from defective viruses against which a CTL response could be primed anyway. However, it can neither be ruled out that the experimental assessment led to a false classification of an AAS by affecting binding of the antibodies used in the ELISA analysis^60^. Alternatively, some mutations causing a fitness defect could have been present in a Gag-variant also harboring a compensatory mutation, as has for instance been reported for the HLA-B27-restricted KK10 epitope^61^. Given such possible interactions it would be interesting to perform similar classifications for AAS-combinations. However, the theoretically possible number of such combinations that would have to be computationally analysed is very large and prohibits such an approach. A realizable alternative could be to analyse only pair-wise combinations, which would be possible for AAS numbers that can be reasonably expected (currently 158). For such an extension, however, it would be necessary to modify the classifier by performing a training on a set of variants with known budding phenotype that harbor AAS combinations. Yet, the current approach already yielded very good results, with all of the three TeeGags that were experimentally assessed being budding-competent. In addition, an up-front curation step that manually excludes AAS known to interfere with budding based on literature-data could further improve the realization probability of budding-competent TeeGag-antigens.

By combining iteratively generated TeeGags into multivalent Gag-antigens quite a significant coverage of the set of known CD8^+^ T cell epitopes can be achieved (Fig. 3). However, it remains to be demonstrated that application of TeeGag antigens, either delivered as recombinantly produced VLPs or produced *in vivo* after delivery as a DNA-vaccine, or using viral vectors, elicits broader T cell responses. As the design was directed towards incorporating epitopes restricted by human MHC molecules, assessment in animal models (mice, non-human-primates) is difficult. Therefore, analyses in human *ex vivo* assays or possibly also models of humanized mice will be required to determine whether the TeeGags are superior antigens in regard to eliciting broadly reactive T cell responses. In conclusion, only via an extensive immunogenicity analysis with special emphasis on human MHC-directed epitope-presentation, it will eventually be possible to verify whether the described design strategy translates into improved immune responses.

Since efficient priming of CD8^+^ T cells requires additional help by CD4^+^ T cells that recognize related antigens on the same APC, improved presentation of MHC-II-restricted T cell epitopes might be beneficial. In this regard, the algorithm also could easily be applied to enrich MHC-II-restricted epitopes by including the set of CD4^+^ T cell epitopes from the LANL database (currently about 400 entries) and amending the scoring function for a user-adjustable parameter to control the balance between MHC-I- and MHC-II-restricted epitopes. Incorporation of linear B-cell epitopes would also be possible, though this is likely not relevant for Gag, but could rather be used for the generation of a likewise TeeEnv antigen. However, it must be considered that B cell epitopes, especially those recognized by broadly-neutralizing antibodies, are often structural determinants that cannot be treated like linear T cell epitopes. However, if the residues contributing to the antibody-epitope are known, these can be kept constant, and T cell determinants could be enriched in the remaining sequence parts. Finally, otherwise designed Env-antigens retaining the transmembrane-domain could be incorporated into Gag-VLPs built from TeeGags. Such complexes might prove to be very useful immunogens, as they would deliver (i) TeeGag for elicitation of CD4^+^ and CD8^+^ T cell responses, (ii) Env for elicitation of antibody responses, and (iii) the physical combination in a VLP might direct CD4 T cells toward the Env-specific B cells by intrastructural help^62,63^.

## Material and Methods

### Data sources

The premade filtered web-alignment of Gag (2013) was downloaded from the LANL database (http://www.hiv.lanl.gov/content/sequence/NEWALIGN/align.html) and curated by excluding non-M-group and unclassified (“U”) sequences (84 of 5085). Consensus and ancestral Gag sequences (2004) were obtained from LANL, mosaic sequences from^18^. Center-of-tree sequences were kindly provided by James Mullins.

CD8^+^ T cell epitopes were retrieved from LANL’s HIV molecular immunology database on April 24, 2015 using the search parameters: HIV protein = p17, or p24, or p2p7p1p6; species = human. The following properties were derived from the database’s annotation fields for scoring: HLA-association (database’s MHC/HLA-field), epitope’s viral subtype origin (subtype-field), association with LTNP and experimentally determined fraction of responders (manually retrieved from notes-field).

### Classifier

#### Structure-based classification feature

Initially, HXB2 reference 3D-structures for the individual Gag domains p17, p24, p2, p7, and p6 were built by homology modelling using the automodel function of MODELLER version 9.7^38^ using PDB structures as templates (1L6N + 1TAM for p17; 3H47 + 3GV2 + 2K1C for p24; 1U57 for p2; 1AAF for p7; 2C55 for p6). To analyze the impact of an AAS, a 3D structure of Gag containing the respective AAS was modelled accordingly. Next, a per residue energy landscape profile was calculated by using the DOPE model^37^. Four different scores were then derived: (i) the DOPE free energy value of the AAS (local DOPE), (ii) the DOPE-value-difference between the reference and mutant structure at the AAS’s position (local ΔDOPE), (iii) this difference summed up for all aa (global ΔDOPE), and (iv) the difference summed up from −6 to +5 aa around the AAS (window ΔDOPE).

#### Sequence-based classification feature

A position-specific-substitution matrix (PSSM) over the length of Gag for all amino acids was built (500 × 20 matrix). First, observed frequencies *f*_*ka*_ of the occurrence of all amino acids *a* at each position *k* were calculated from the curated Gag alignment. To account for unobserved substitutions, artificial pseudo-counts were added with frequencies of *b*_*ka*_. These pseudo-frequencies were calculated by conditioning the general mutation probabilities *q*_*ia*_ for substitution of amino acid *a* by *i* (taken from^64^) with the observed frequency as

1 $${b}_{ka}=\sum _{i}({f}_{ki}\cdot \frac{{q}_{ia}}{\sum _{a}{q}_{ia}})$$

Accounting for the number of sequences in the alignment (*N*_*k*_), and the number of added pseudo-counts (*B*_*k*_), the probability *p*_*ka*_ of *a* occurring at *k* is estimated as

2 $${{\rm{p}}}_{{\rm{ka}}}=\frac{{{\rm{N}}}_{{\rm{k}}}}{{{\rm{N}}}_{{\rm{k}}}+{{\rm{B}}}_{{\rm{k}}}}\cdot {{\rm{f}}}_{{\rm{ka}}}+\frac{{{\rm{B}}}_{{\rm{k}}}}{{{\rm{N}}}_{{\rm{k}}}+{{\rm{B}}}_{{\rm{k}}}}\cdot {{\rm{b}}}_{{\rm{ka}}}$$

*B*_*k*_ was calculated as *m* × *R*_*k*_, where *R*_*k*_ is the number of different aa at *k*, weighted by an empirical number *m* (chosen as *m* = 5 as recommended in^65^). Finally, the PSSM score *w*_*ka*_ was calculated as log-odds-ratio of *p*_*ka*_ and the background frequency *p*_*a*_ of *a* (obtained from http://www.pseudogene.org/composition/index.cgi):

3 $${{\rm{w}}}_{{\rm{ka}}}=\,\mathrm{log}(\frac{{{\rm{p}}}_{{\rm{ka}}}}{{{\rm{p}}}_{{\rm{a}}}})$$

Thus, the score for the sequence-based feature of an AAS can directly be taken from the *w*_*ka*_-PSSM.

#### Classification

Discriminatory capacity of individual features was assessed by grouping the training-set AAS according to budding-compatibility and comparing the scores with a Wilcoxon rank sum test in R (version 3.0.2; The R Foundation for Statistical Computing). For the classification of AAS with unknown effect on budding, a trained classifier was implemented using Fisher’s linear discriminant analysis^41^. 100 repeats with 10-fold cross-validation were performed and the test characteristics for each feature combination were calculated (see Supplementary Table S2).

### Scoring

Scores representing immunological and virological properties of CD8^+^ T cell epitopes were calculated by evaluating the epitope’s metadata. If applicable, annotations of superepitopes were amended by annotations of the corresponding subepitopes.

As HLA-association is heterogeneously given either for the allele, or only the allele group, all annotations were harmonized to allele groups (e.g. HLA*A2:01:01 to HLA*A2). The HLA-association-score *s*_*h*_*(e)* of epitope *e* was calculated by summing up the population frequencies (from^43^) of all associated allele groups.

The viral-subtype-score *s*_*s*_*(e)* is calculated by summing up user-supplied weighting parameters *w*_*s*_ for the 12 individual clades (A, B, C, D, F, G, H, J, K, CRF01_AE, CRF02_AG, CRF07_BC) if the epitope is derived from a virus of the respective clade. Here, the world-wide frequencies of clades circulating between 2004 and 2007^44^ were chosen as weights (*w*_*s*_).

If an epitope’s description stated that it was identified in an LTNP, the epitope received an LTNP-score *s*_*l*_*(e)* of 1, otherwise none.

If the metadata contained information on the fraction of people from patient cohorts with responses toward a given epitope, this fraction *f(e)* was taken as the experimental-recognition score *s*_*r*_*(e)*. If the cohort was pre-selected regarding carrier-status of a certain HLA-allele, the allele frequency *h* was factored in:

4 $${{\rm{s}}}_{{\rm{r}}}({\rm{e}})={\rm{f}}({\rm{e}})\cdot (2{\rm{h}}-{{\rm{h}}}^{2})$$

Conservation of an epitope was first calculated per subtype as frequency *f*_*s*_*(e)*, representing the fraction of sequences from the Gag alignment of each subtype *s* harboring the exact epitope sequence. The overall conservation score *s*_*c*_*(e)* was then calculated by summing up the weighted frequencies:

5 $${{\rm{s}}}_{{\rm{c}}}({\rm{e}})=\sum _{{\rm{s}}}({{\rm{w}}}_{{\rm{s}}}\cdot {{\rm{f}}}_{{\rm{s}}}({\rm{e}}))$$

The overall score *s(e)* of epitope *e* was eventually calculated as the sum of the normalized individual scores *s*_*k*_*(e)* outlined above (*k* = *h, s, l, r, c*), taking user-defined weighting parameters *w*_*k*_ into account:

6 $${\rm{s}}({\rm{e}})=\sum _{{\rm{k}}}({{\rm{w}}}_{{\rm{k}}}\cdot {{\rm{s}}}_{{\rm{k}}}({\rm{e}}))$$

### Antigen Assembly

Many epitopes span overlapping parts of the sequence. If two epitopes harbor different AAS in the overlap, such epitopes are mutually exclusive for incorporation into the same antigen sequence (“incompatible epitopes”). To select a compatible set, an incompatibility graph was initially created, in which vertices represent epitopes, with the weight *w*_*i*_ of vertex *i* given by the epitope’s score *s(e)*, and with edges connecting vertices of incompatible epitopes. A genetic algorithm (GA) was used to select an epitope set that approximates the theoretically best solution, i.e. as many and as high-scoring epitopes as possible. The GA was initialized with a population of 70 chromosomes *x* that each represent a bit vector of length *v* for all vertices. Each bit *x*_*i*_ indicates the presence (1) or absence (0) of vertex *i*. Initially, the chromosomes were populated with random, unique selections of compatible epitopes, i.e. non-connected vertices. The fitness of the chromosomes was calculated by the fitness function

7 $$f(x)={(\sum _{i=1}^{v}({x}_{i}\cdot {w}_{i}))}^{2}+20\cdot {n}_{{\rm{\sup }}(x)}+{n}_{sub}(x)$$

where *n*_*sup*_ and *n*_*sub*_ are the numbers of superepitopes and subepitopes, respectively. The chromosome with the highest fitness value represents the current “solution representative”.

In each evolution round, 24 chromosomes were generated and populated with newly initialized random chromosomes to maintain genetic diversity (probability 0.05), or by generating offspring as follows: (1) Selecting two chromosomes with a probability proportional to their fitness. (2) Replacing one with a newly initialized chromosome with probability 0.4. (3) Generating offspring chromosome by three-point crossover at random sites, restricted in a way that the offspring receives numbers of bits relative to the parents’ fitness. (4) Spontaneously mutating each chromosome by flipping random bits with probability 0.01. If the chromosome harbored incompatible epitopes (connected vertices), the offspring was repaired by repeatedly setting the bit with highest number of incompatibilities to 0 until all incompatibilities were resolved. Chromosomes in the subpopulation already contained in the overall population were rejected. Finally, offspring chromosomes were each compared to four randomly selected chromosomes from the parent population, and replaced the one with the lowest fitness, if the offspring scored higher. In total, 2000 rounds of this process were executed and the final solution representative was chosen as antigen candidate. Positions not covered by an epitope were filled in with the respective aa from HXB2. Then, all epitopes contained in this candidate were removed from the incompatibility graph and the GA was repeated with the reduced epitope set to come up with a second solution representative as complementary antigen candidate. This overall process was repeated until all epitopes were covered in the set of complementary antigens.

### *In-silico* Analysis

To generally compare Gag sequences by computational methods, the numbers of epitopes from the input set contained within the respective sequences were counted. In addition, the scores *s(e)* of these epitopes were calculated and summed up to assign an overall score-parameter to every Gag-sequence. To avoid overestimations of epitope scores, subepitopes were not considered if a corresponding superepitope was present in the sequence.

The population score was calculated by first simulating HLA haplotypes of 1,000 members of the world-wide general population by randomly picking two HLA-A, -B, and -C allele groups with a probability matching the natural allele frequencies^43^. Next, only if an epitope was known to be restricted by at least one allele, it was retained. The population coverage and score resemble the average number and overall score of retained epitopes.

The pathogen coverage and score were calculated as average from 1,000 iterations of the following procedure: The sequences of the Gag alignment were split into clade-specific subsets. Then, a clade was randomly chosen with a probability resembling its global frequency^44^ and one sequence of this subset was selected. The number and overall score of epitopes in this sequence matching those present in the Gag sequence of interest were finally calculated.

### Experimental Analysis

Human codon-optimized DNA sequences encoding the Gag-variants were obtained from GeneArt (Thermo Fisher Scientific, Germany) and cloned into pcDNA3.1(+) via KpnI and XhoI sites. Gag variants with single AASs were generated by PCR-mediated directed mutagenesis according to standard procedures.

Human embryonal kidney 293 T cells (ATCC-# CRL-11268) were cultivated in DMEM medium with 10% FCS and 1% Pen/Strep at 37 °C and 5% CO_2_. The day before the transfection, 1.4 × 10^5^ cells in 1 ml were seeded per well of a twelve-well plate. Cells were co-transfected with 1 µg of the respective Gag-plasmid and 0.05 µg of the CMV-SEAP-plasmid (Addgene #24595, kind gift from Alan Cochrane) using PEI in a 1:5 (w/w) ratio^66^. Medium was replaced 6 h later by DMEM_0_. Conditioned media with Gag virus-like particles were harvested after 48 h, cleared by centrifugation and treated with 0.5% TX-100. The Gag content was quantified via a p24-ELISA as described in^67^. SEAP-activity was assayed colorimetrically as described in^68^.

### Data availability

All data generated or analysed during this study are included in this published article and its Supplementary Information files.

## Electronic supplementary material

Supplementary Information

Supplementary Dataset 1

Supplementary Dataset 2

Supplementary Dataset 3
