# Supplementary material for: Computational Design of Epitope-Enriched HIV-1 Gag Antigens with Preserved Structure and Function for Induction of Broad CD8+ T Cell Responses
Source: Sci Rep. 2018 Jul 26;8:11264. doi: 10.1038/s41598-018-29435-1 (PMC6062507; doi:10.1038/s41598-018-29435-1)
Supplement: Supplementary file 1 — Supplementary Information [file 41598_2018_29435_MOESM1_ESM.pdf]

Supplementary Information

Computational Design of Epitope-Enriched HIV-1 Gag Antigens with Preserved Structure and Function for Induction of Broad CD8$^+$ T Cell Responses

Benedikt Asbach[1¶], Johannes P. Meier[*1¶], Matthias Pfeifer[1], Josef Köstler[2], Ralf Wagner[1,2*]

[1] Molecular Microbiology (Virology), Institute of Medical Microbiology and Hygiene, Universität Regensburg, Regensburg, Germany

[2] Institute of Clinical Microbiology and Hygiene, Universität Regensburg, Regensburg, Germany

* Corresponding author
E-mail: ralf.wagner@ur.de

¶ These authors contributed equally to this work, in alphabetical order

Table S1: Number of epitopes (and score in %) covered in various Gag-sequence-designs. (n.a. = not available)

	Consensus	Ancestral	Center-of-tree	Mosaic
Clade A1	238 (33.5 %)	250 (35.9 %)	n.a.	n.a.
Clade B	401 (52.0 %)	352 (44.6 %)	393 (51.5 %)	400 (52.0 %)
Clade C	271 (45.3 %)	269 (41.2 %)	275 (46.1 %)	275 (45.8 %)

Table S2: Formulae for calculation of test characteristics
TP = number of true positives, TN = number of true negatives, FP = number of false positives, FN = number of false negatives

Test characteristic	Formula
Accuracy	$\frac{TP + TN}{TP + FN + TN + FP}$
Precision	$\frac{TP}{TP + FP}$
Negative predictive value	$\frac{TN}{TN + FN}$
Sensitivity	$\frac{TP}{TP + FN}$
Specificity	$\frac{TN}{FP + TN}$
Matthews correlation coefficient	$\frac{TP \times TN - FP \times FN}{\sqrt{(TP + FP)(TP + FN)(TN + FP)(TN + FN)}}$

```
HXB2-Gag MGARASVLSGGELDRWEKIRLRPGGKKKYKLKHIVWASRELERFAVNPGL 50
TeeGag1  ..................................................
TeeGag2  ...........Q.................R...L...........L....
TeeGag3  ...........K..S............H.M...L................

HXB2-Gag LETSEGCRQILGQLQPSLQTGSEELRSLYNTVATLYCVHQRIEIKDTKEA 100
TeeGag1  ..................................................
TeeGag2  .......K.............T.....................V......
TeeGag3  ..S......................K..............K.D.......

HXB2-Gag LDKIEEEQNKSKKKAQQAAADTGHSNQVSQNYPIVQNIQGQMVHQAISPR 150
TeeGag1  .E............T......K.N.S...........L............
TeeGag2  .........................GK..........L............
TeeGag3  ........................GKK..................SL...

HXB2-Gag TLNAWVKVVEEKAFSPEVIPMFSALSEGATPQDLNTMLNTVGGHQAAMQM 200
TeeGag1  ........I.........................................
TeeGag2  ......................T............M...I..........
TeeGag3  ........I.............T...........................

HXB2-Gag LKETINEEAAEWDRVHPVHAGPIAPGQMREPRGSDIAGTTSTLQEQIGWM 250
TeeGag1  ..............L...................................
TeeGag2  ..D............................................A..
TeeGag3  ......................V.......................VA..

HXB2-Gag TNNPPIPVGEIYKRWIILGLNKIVRMYSPTSILDIRQGPKEPFRDYVDRF 300
TeeGag1  ..................................................
TeeGag2  .S.......D...................V.....K..............
TeeGag3  .S...V...D.......M...........V....................

HXB2-Gag YKTLRAEQASQEVKNWMTETLLVQNANPDCKTILKALGPAATLEEMMTAC 350
TeeGag1  ..................................R....G..........
TeeGag2  F........T.D......D...............................
TeeGag3  ...........D.............S........R....G.S........

HXB2-Gag QGVGGPGHKARVLAEAMSQVTNSATIMMQRGNFRNQRKIVKCFNCGKEGH 400
TeeGag1  ..............................S..KGNKRM...........
TeeGag2  ......S............ANSA............SKR............
TeeGag3  ......S....I............N.............T.......R...

HXB2-Gag TARNCRAPRKKGCWKCGKEGHQMKDCTERQANFLGKIWPSYKGRPGNFLQ 450
TeeGag1  I.K...............................................
TeeGag2  I.........................N.......................
TeeGag3  ..................................................

HXB2-Gag SRPEPTAPPEESFRSGVETTTPPQKQEPIDKELYPLTSLRSLFGNDPSSQ 500
TeeGag1  .........A....FEET.PA.K.............A.............
TeeGag2  .........F....F.E.....S.............A..K..........
TeeGag3  ..............F.E.....S....Q...........K....S.....
```

Figure S1: Alignment of TeeGag1, TeeGag2, and TeeGag3 protein sequences. Positions identical to the reference Gag-sequence of the HXB2-isolate are denoted with ".", otherwise the respective amino acid is given.

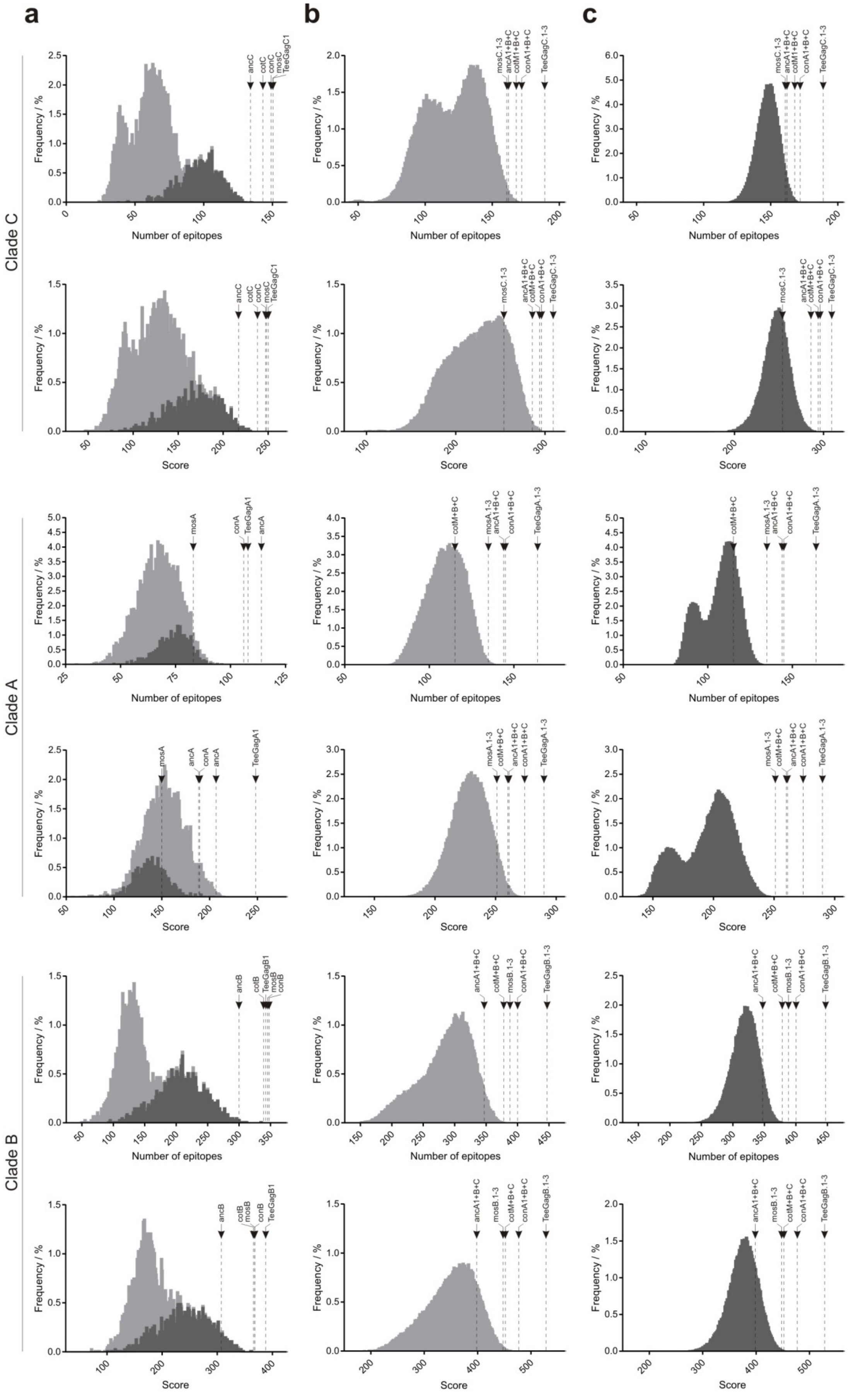

Figure S2: Comparison of epitope coverage and score of clade-specific

artificial designs.

Clade-specific TeeGag sequences were generated (see S3 dataset) for the three most frequent HIV clades, i.e. C, A, and B using only epitopes associated with the respective clade in the input set and disabled subtype weighting in the scoring function. The sequences were assessed regarding epitope coverage and score as in figure 5 for epitopes only derived from the respective clade indicated on the left. (a) Values for monovalent sequences; natural sequences from the clade indicated on the left are shown in dark grey, all other natural sequences in light grey. (b) Values for trivalent antigen combinations compared to trivalent combinations of natural sequences from any clade (light grey) or (c) only from the clade indicated on the left. ancA and conA refer to subclade A1 only. The mosA sequence was generated using the Mosaic Vaccine Designer at LANL using the clade A Gag sequences from the filtered web alignment as input, with the following parameters: Cocktail Size = 1 for monovalent, or 3 for trivalent set; Epitope Length = 9; Rare Threshold = 3.

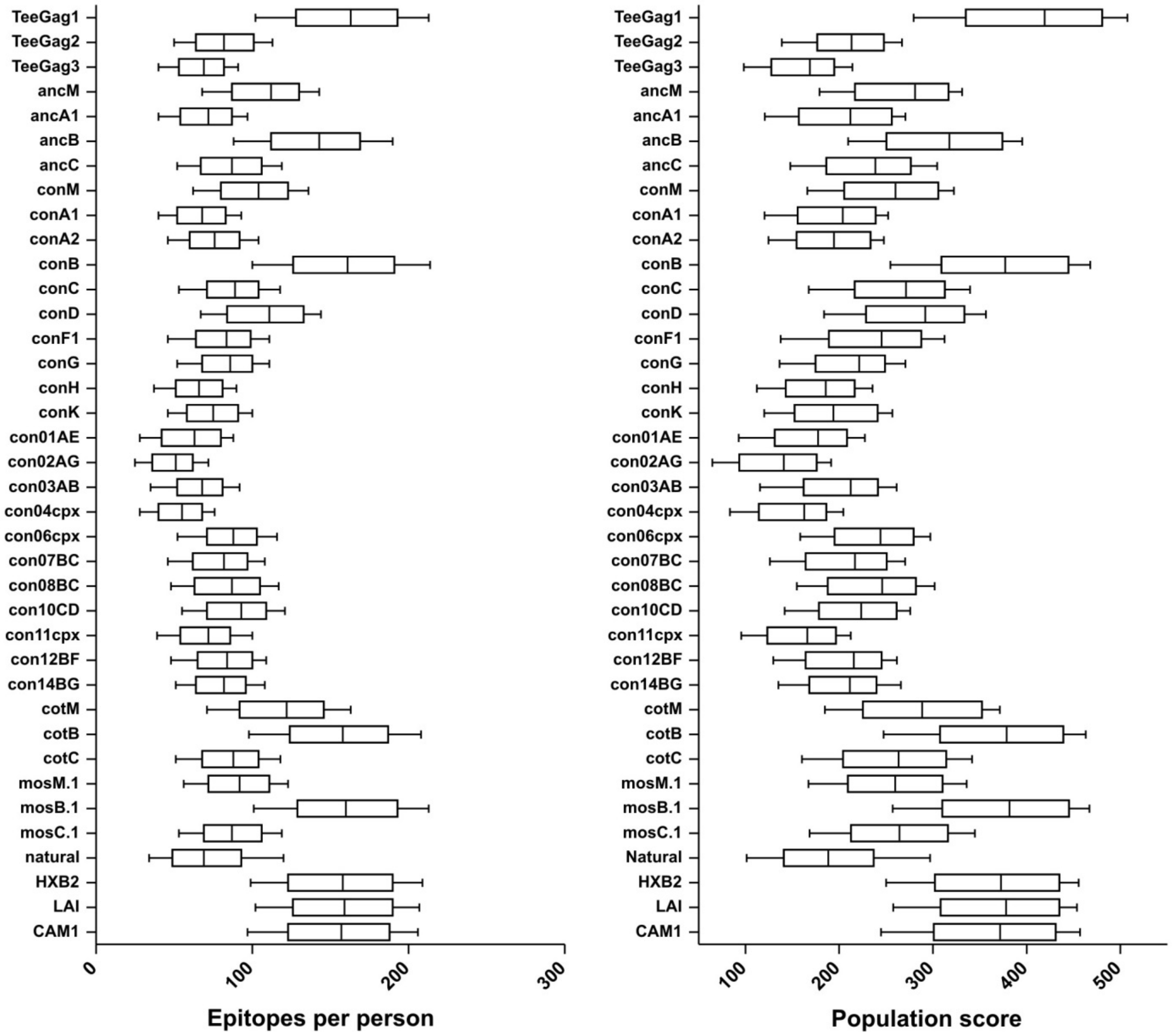

Figure S3: Population coverage for additional Gag designs and natural Gag isolates.

The number of epitopes (left panel), and the overall scores (population-score, right panel) for monovalent M-group- and clade-specific ancestral (anc), consensus (con), center-of-tree (cot), and mosaic (mos) Gag designs, as well as for natural Gag isolates, were determined for 1000 HLA haplotypes that were randomly generated by selecting two HLA-A, -B, and -C alleles each, respecting the allele frequencies. Boxes show the median and 50% quartiles, whiskers the 10 and 90% percentiles.

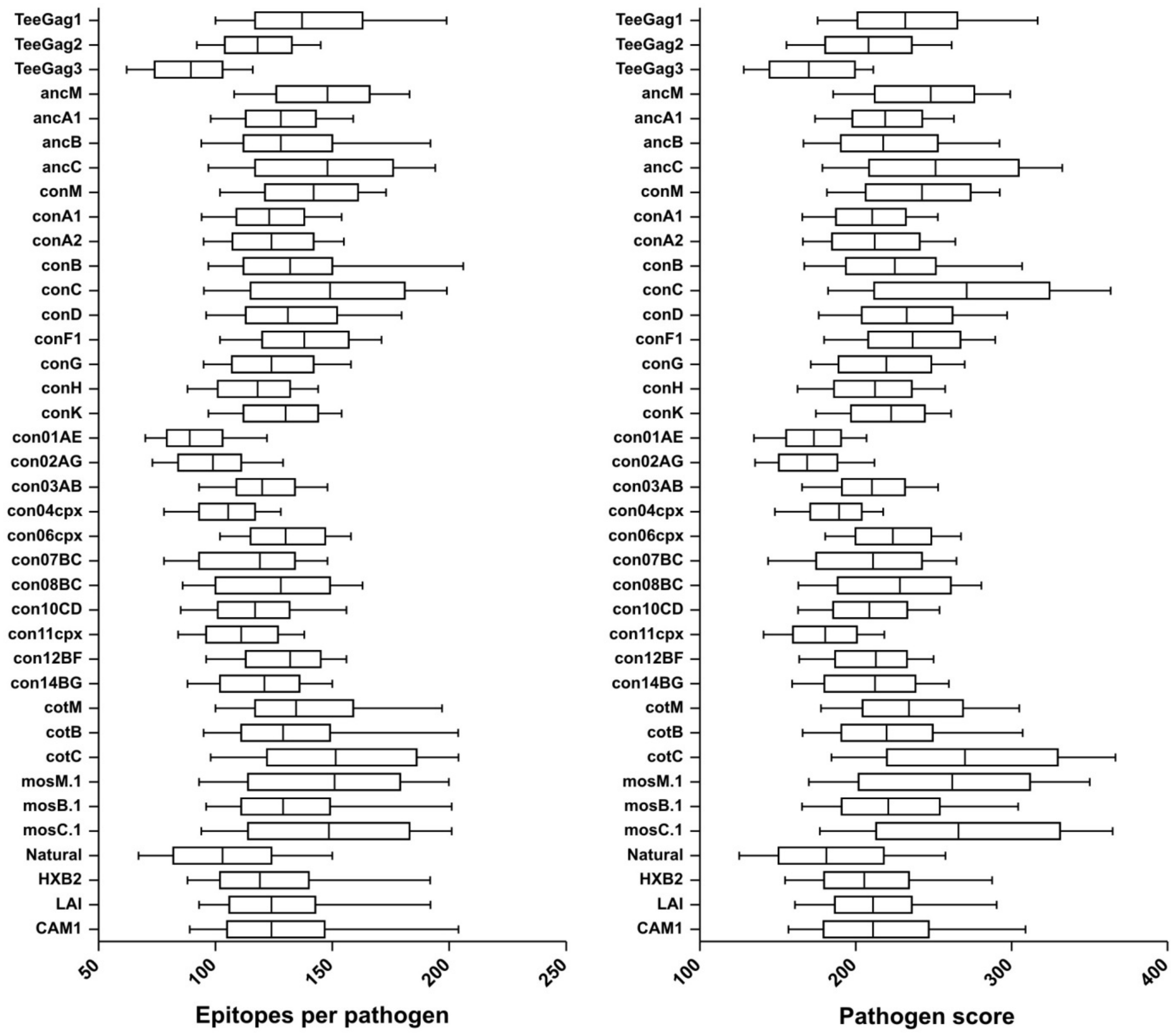

Figure S4: Pathogen coverage for additional Gag designs and natural Gag isolates.

The number of epitopes (left panel), and their summed up scores (pathogen-score, right panel) for monovalent M-group- and clade-specific ancestral (anc), consensus (con), center-of-tree (cot), and mosaic (mos) Gag designs, as well as for natural Gag isolates, were determined for 1000 randomly picked natural Gag-isolates (pathogens), respecting the clade-frequencies. Boxes show the median and 50% quartiles, whiskers the 10 and 90% percentiles.
